# Nomophobia, phubbing and social phobia in Portuguese young adults and adults

**DOI:** 10.1192/j.eurpsy.2024.983

**Published:** 2024-08-27

**Authors:** B. R. Maia, D. Sousa

**Affiliations:** ^1^Universidade Católica Portuguesa; Center for Philosophical and Humanistic Studies; ^2^Universidade Católica Portuguesa, Braga, Portugal

## Abstract

**Introduction:**

To our knowledge there is no study exploring the interrelationship between nomophobia, phubbing and social phobia in Portuguese young adults and adults.

**Objectives:**

To explore the nomophobia, phubbing and social phobia levels, the interrelationship between these three constructs, in a sample of Portuguese young adults and adults.

**Methods:**

316 subjects, with a mean age of 25.71 years old (*SD* = 8.231; range 18 - 59) fulfilled a sociodemographic questionnaire, and the Portuguese validations of the Nomophobia Questionnaire, the Phubbing Scale and the Social Interaction and Performance Anxiety and Avoidance Scale.

**Results:**

All the subjects presented nomophobia (100%, *n* = 316), with 62% (*n* = 196) presenting a moderate risk level and 22% (*n* = 69) an higher risk level. The mean of the ‘total phubbing score’ was of 21.50 (*DP* = 5.50) and ‘smartphone obsession’ was the phubbing subscale with an higher score (*X* = 12.81, *DP* = 3.50). The mean of the total nomophobia was of 80.0 (DP = 22.83) and ‘not being able to communicate’ was the nomophobia subescale with an higher score (*X* = 24.75, *DP* = 9.95).Considering social phobia scale, the mean of the ‘anxiety/distress’ subscale was of 95.36 (*DP* = 25.14) and of the ‘avoidance subscale’ was of 89.56 (*DP* = 25.53). Almost 22% (*n* = 69) of the subjects presented ‘social anxiety’ and 24% (*n* = 76) presented ‘social avoidance’, suggesting probable social phobia cases (higher than the proposed cut-off scores). Positive and significant correlations were found between all the nomophobia and phubbing subscales (ranging from .30** to .61**). Positive and significantly correlations, mostly with low magnitude, were found between nomophobia and social phobia subscales (ranging from .03** to .22**), except for ‘social avoidance’ subescale, which correlation was negative (-.021*). Females presented higher levels of nomophobia (*Md* = 176.28) and phubbing (Md = 167.22) than males (*Md* =124.73, *U* = 7301.500, *p* <.001;*Md* = 141.93), *U* = 9475.500, p= .019, respectively). Total social phobia scores and nomophobia (not being able to access information and giving up convenience subescales) were significantly higher in young adults.

**Conclusions:**

Nomophobia, phubbing and social phobia are significantly intercorrelated. Future longitudinal studies are needed to clarify nomophobia and phubbing etiology. The level of nomophobia (100%) found in this sample is specially worrying.

**Disclosure of Interest:**

None Declared

